# The effect of management control systems in managing the unknown: Does the market appreciate the breadth of vision?

**DOI:** 10.1007/s11846-022-00601-0

**Published:** 2022-11-28

**Authors:** Jacobo Gomez-Conde, Ernesto Lopez-Valeiras, Fabricia Silva Rosa, Rogério João Lunkes

**Affiliations:** 1 Departamento de Contabilidad, Universidad Autónoma de Madrid, Francisco Tomás y Valiente, 5, 28049 Madrid, Spain; 2 ECOBAS, Universidad de Vigo, Departamento de Economía Financeira e Contabilidade, Ourense, Spain; 3 Departamento de Ciências Contábeis, Universidade Federal de Santa Catarina, Florianópolis, Brazil; 4 Biocost Research Group, Galicia Sur Health Research Institute (IIS Galicia Sur), SERGAS-UVIGO, Pontevedra, Spain

**Keywords:** Management control systems, Management accounting, Broad scope, COVID-19, Crisis management, Signaling theory, 62P20, 91B82

## Abstract

We examine the extent to which broad-scope management control systems (MCS) mitigate the negative impact of a crisis with extreme uncertainty on investor and shareholder expectations and the potential role of boundary systems in this link. We use the COVID-19 pandemic as research setting to analyse this link and market value as a proxy for expectations. Our hypotheses are tested using a combination of survey and archival data from large organizations listed on the Brazilian Stock Exchange, resulting in a panel of 6257 organization-week observations. Our main results are consistent with the hypotheses. We also conduct a series of sensitivity tests to check the robustness of our main findings. Our results remain significant across specifications: alternative identification strategy, or additional control variables. In an additional analysis, we also examine the role of lenders. Overall, we extend the scarce prior literature on the effectiveness of MCS under crisis management and provide new evidence for signaling theory, thus connecting both streams of literature. The COVID-19 pandemic provides an optimal context for researching this topic because, in contrast to past economic downturns or financial crises, it has required organizations across industries to adapt quickly and respond to new demands with unpredictable economic, behavioural, and societal consequences.

## Introduction

We empirically examine the extent to which broad-scope management control systems (MCS) mitigate the negative impact of the COVID-19 pandemic on investors and shareholders’ expectations, as captured by market value. The intuition behind this hypothesis is that stock markets react to news and organizational signals, particularly in emerging markets (Morck et al. 2000). We argue that managers using MCS designs that are broad rather than narrow in scope have access to more information that facilitates more effective managerial decision-making when managing complex and competing forces from the external environment (Jordao et al. 2014; Nielsen et al. 2015). By increasing the breadth of information, numbers and calculations that managers have access to, broad-scope MCS can enhance managers’ *manoeuvres* to adapt rapidly to new market situations and decrease an organization’s exposure to environmental uncertainty (Tillema 2005; Naranjo-Gil and Hartmann 2007). According to signaling theory, organizations voluntarily disclose information about their actions to minimize the asymmetry of information with investors and analysts (Alsos and Ljunggren 2017; Callen et al. 2016). The disclosure of broad and future-oriented figures (e.g., projections and segmented information) may provide markets with new information not available elsewhere. Investors and analysts have great resources at their disposal to capture the signals and analyse organizations’ strategic actions.^1^ Consequently, share prices quickly incorporate new information. As investors value the ability of organizations to move rapidly in the market, they adjust their judgements and increase their expectations about the quality of organizations’ future performance. Prior work recognizes that growth expectations constitute key information that stock markets appreciate (Angulo-Ruiz et al. 2018; Jain et al. 2016).

We suggest that in major crises and unknown situations, such as the unprecedented and catastrophic COVID-19 pandemic (Rinaldi et al. 2020; Navarro-Picado et al. 2022), organizations equipped with broad-scope MCS are able to make better and informed decisions (Kober and Thambar 2021), and their strategic market movements are detected by investors. Interestingly, despite the potential for MCS to influence and support managers’ forward-looking decision-making, there is scant empirical evidence on their association with market reactions in the accounting and finance literature, even though prior work recognizes the usefulness and effectiveness of MCS for organizations that perceive high levels of environmental uncertainty (Otley 2016; Guenther and Heinicke 2019; Sageder and Feldbauer-Durstmüller 2019; ten Rouwelaar et al. 2021). Signaling theory also suggests that markets quickly incorporate these movements and new information about organizations with more broad-scope MCS, which might be better able to identify new opportunities. We empirically analyse this hypothesis in our paper.

We also argue that, in this setting of high uncertainty, a clear delineation of tolerable decisions and opportunity-seeking behaviours is a question of interest. Since managers are trying to increase the usefulness of their responses to the crisis, we study whether firms with a high delineation of boundary systems are more likely to make within-bound decisions and actions and consequently obtain better market returns.

We test our hypotheses using a combination of archival and survey data gathered from 168 organizations listed on the Brazilian Stock Exchange (B3). Our empirical analysis, which uses a sample of 6257 week-organization observations from July 2019 to July 2020, indicates that the results are consistent with our expectations. In particular, we find that organizations that demonstrate greater use of broad-scope MCS have higher market value to EBITDA after the World Health Organization (WHO) declared COVID-19 a worldwide pandemic. According to our expectations, the results suggest that the positive effect of broad-scope MCS under COVID-19 is concentrated in firms with higher levels of boundary systems. These results remain stable under a variety of sensitivity and robustness checks.

The contribution of this research is twofold. First, this study offers further evidence of how managers, through internal business practices, signal information to markets. Most accounting literature has focused on the signals that organizations send to the market via financial structure and dividends, disclosures and assurance practices (e.g., Callen et al. 2016; Jain et al. 2016; Pham et al. 2019; Dordzhieva et al. 2022). However, little is known about the extent to which MCS designs could also reflect on investor perceptions (Davila et al. 2015). Second, this study extends the scarce prior literature on the role of MCS in crisis management (Becker et al. 2016). While most management control literature has focused on perceived environmental uncertainty (e.g., King et al. 2010), economic crisis perception (e.g., Asel et al. 2011; Janke et al. 2014) or organization (sector)-specific crises (e.g., Conrad and Guven-Uslu 2012), our research offers evidence of the extent to which specific MCS designs assist firms during a major worldwide pandemic. In addition, we approach this issue using a combination of archival and survey measures.

The remainder of this paper is structured as follows. Section two provides the theoretical background and develops the argumentation leading to our hypotheses. Section three outlines the empirical design, while section four presents our main and additional findings. Finally, section five discusses the results and concludes the paper.

## Prior literature and hypothesis development

### Signaling theory

Signaling theory has gained prominence in prior literature analysing market reactions and investment decisions (Alsos and Ljunggren 2017; Dordzhieva et al. 2022). This theory was developed to understand how parties interpret and respond in settings where information is asymmetrically distributed. The core assumption is that signalers are insiders who have access to data and information not accessible to receivers who are outsiders (Bergh and Gibbons 2011; Arzubiaga et al. 2022). In other words, managers have information that investors do not. Signaling theory indicates that the information asymmetry between organizations and investors can be reduced when organizations provide high-quality information (not accessible by other means) as a signal to the market (Callen et al. 2016; Dordzhieva et al. 2022). Better disclosure, informing managers about plans for the future, increases management's credibility in the eyes of the market (Eccles et al. 2001; Bilinski 2022). These signals are a credible form of communication and allow recipients (e.g., investors) to differentiate between organizations (Florio and Leoni 2017). Nondisclosure of this future-oriented information can be viewed as a signal of ‘bad’ news (Watson et al. 2002).

The pandemic thoroughly altered many dimensions of organizational managerial practices and capital market dynamics (Bouncken et al. 2022), including corporate communication and the use of communication channels to send signals (Wendt et al. 2021).^2^ Managers work hard to distinguish themselves from ‘lower quality firms’ through voluntary disclosures using these new communication channels together with more traditional channels (e.g., interim reports). In this regard, Watson et al. (2002), focusing on the disclosure of accounting ratios, suggest that “for managers to signal quality successfully, the signal must be credible. In this case, credibility is achieved as ultimately the true quality of the firm will be verifiable. If managers falsely try to signal that they are of high quality, when in fact they are of low, once this has been revealed, no subsequent disclosures will be seen as credible” (p. 291). In turn, the penalty can be even higher.

We build on signaling theory to predict how the stock market reacts to the strategic movements of organizations using broad-scope MCS. Uncertainty and information asymmetry surrounding firms’ future under a major crisis make it difficult for investors to calculate earnings prospects (Zhang and Wiersema 2009). Consequently, they trust firm movements derived from ‘signals’ by which to differentiate the quality of organizations. In a management control context, organizations send signals to investors about future prospects, such as their ability to earn future positive cash flows (Davila et al. 2015; Jain et al. 2016). Recent case studies in the context of the pandemic (e.g., Kober and Tambar 2021) also find that the availability of externally focused information supports managers in times of high uncertainty to determine how to respond to immediate business challenges and plan for the *postcrisis* world. Additionally, there is evidence of the critical role of using some broad-scope indicators to provide a compass for decision-making during COVID-19 (McKinsey and Company 2020; Kober and Tambar 2022).^3^ With broad-scope MCS, organizations are able to quickly detect market changes and anticipate decisions to enter new market segments, to shift towards more digital business models, or to offer new products to customers that they already have in their portfolio. These strategic organizational decisions are signals that managers send to investors to reduce asymmetries.

### Management control systems and informational characteristics

We follow the well-known work of Anthony (1965) to define MCS as “the process by which managers assure that resources are obtained and used effectively and efficiently in the accomplishment of the organization’s objectives” (p. 17). This definition is in line with the definitions subsequently developed, which include financial, strategic and operational metrics and information to improve decision-making and planning (Bisbe and Malagueño 2012; Nielsen et al. 2015; de Harlez and Malagueño 2016; Gomez-Conde et al. 2022).

The design of MCS shapes the characteristics of the information reported for decision-making (Bisbe and Malagueño 2009; Anzilago et al. 2022). Prior work identifies four main dimensions of information that influence the level of MCS design sophistication (Chenhall and Morris 1986; Abernethy and Brownell 1999; Tillema 2005; Garcia Osma et al. 2018): (i) timeliness, (ii) aggregation, (iii) integration, and (iv) scope. *Timeliness* refers to the frequency and speed of reporting of systematically collected data. *Aggregation* refers to the capacity of MCS to provide information in various comprehensive forms, such as time-period or functional area aggregated data. *Integration* refers to the coordination of various departments and areas and to what extent MCS measures the impact of decisions in one area on operations throughout the organization. *Scope* is usually specified in terms of three dimensions: focus, quantification and time horizon. Thus, broad-scope MCS comprise information that is quantitative and qualitative, internal and external, financial and nonfinancial, and historical and future oriented (Mia and Winata 2008; Nguyen et al. 2017; Anzilago et al. 2022). Those systems include a large spectrum of information that could range, for instance, from product costing to customer satisfaction, from marketing trends to new governmental policies, and from employee training needs to new investment opportunities. In contrast, narrow-scope MCS are based on historical and financial data and on past operations and events within the organization.

While prior work documented the usefulness of these four dimensions of MCS information in different settings, we focus our study on scope. Empirical evidence shows that in environments of high uncertainty, such as an economic crisis, firms need to broaden the focus of their MCS (e.g., Tillema 2005; Naranjo-Gil and Hartmann 2007). The broad scope includes nonfinancial, external and forward-looking information. On the one hand, the availability of this externally focused information supports managers in times of high uncertainty to determine how to respond to immediate business challenges and plan for the postcrisis world (Kober and Tambar 2021). By providing information related to the external environment, such as GDP variations, total sales and market share or changes in consumer habits, this system can indicate the need for drastic changes. For example, it may indicate the need to migrate sales channels from brick-and-mortar stores to online channels. On the other hand, the forward-looking focus (recovery, for example) motivates managers to “begin detailed planning now rather than waiting until it’s clear that the epidemic has passed, and economic activity has restarted. At that point, the firm may be behind the competitors” (McKinsey and Company 2020). Considering the above discussion and drawing on prior work, we focus on scope because it is the dimension that supports managers in navigating a crisis and identifying and embracing opportunities.

### COVID-19 pandemic, the use of broad-scope MCS and the role of boundary systems

The COVID-19 pandemic arose suddenly and unexpectedly and has brought unprecedented pressure and short-term challenges for global business activities (Spicer 2020). Signals of the disruptive consequences were late in arriving at company headquarters due to the extraordinary speed with which the virus spread all over the world. Organizations need to act rapidly on a myriad of issues that include the health and safety of employees and customers, sales, cash flow, supply chain, and marketing. The decision-making process was unstructured, as organizations lacked clear reference points, and historical data were not especially useful. Given the uniqueness and recentness of the COVID-19 pandemic, it is unclear the extent to which prior theoretical or empirical studies can help to predict MCS designs that support managerial decision-making under a new and unknown scenario (Kober and Tambar 2022). Next, we draw on accounting studies on the design of MCS under the uncertainty and crisis management literature to argue that broad-scope MCS may help organizations mitigate the negative effects of the COVID-19 pandemic on investors and shareholders’ expectations.

Previous accounting literature has found that higher levels of perceived environmental uncertainty are positively associated with managers’ increasing interest in external, nonfinancial and ex-ante MCS information for supporting decision-making (Tillema 2005; Naranjo-Gil and Hartmann 2007; Otley 2016). In this vein, previous studies suggest that when facing higher levels of perceived environmental uncertainty, organizations that rely on broad-scope MCS perform better than their counterparts (Agbejule 2005; Nguyen et al. 2017). The underlying assumption of these studies is that broad-scope MCS assist managers in gathering more information that supports more accurate decision-making. Whereas narrow-scope MCS provide limited information in terms of the amount and variety of information available for managers, access to broad-scope information enhances managers’ adaptive capabilities that are needed in dynamic environments (Chenhall and Morris 1986; Bisbe and Malagueño, 2012). Accordingly, studies on the use of MCS information by top management teams found that the use of broad-scope MCS allows them to be better prepared to search the environment for opportunities, uncover emerging needs in the changing market, and respond to evolving environmental demands (Naranjo-Gil and Hartmann 2007; Lopez-Valeiras et al. 2016). However, not all firms use broad-scope MCS, as they have potential costs. Prior work points out that these systems may lead to information overload, being problematic when organizations face external threats (Garcia Osma et al. 2018).

In this study, we postulate that information with a broad scope will be especially necessary and valued by managers, investors and analysts during pandemics such as COVID-19.^4^ Pandemics involve venturing into new contexts, whose complexity and unpredictability make it hard for managers to take action (Bouwens and Abernethy 2000). Crisis management studies indicate that in times of crisis, decision-makers find it difficult to gain access to needed information, which is often difficult to locate and generally complex (König et al. 2020). Under such circumstances, the use of broad-scope MCS could better position managers to respond more quickly to the impact of COVID-19, as they are equipped with more comprehensive information, thereby enabling better and accurate decision-making (Kober and Tambar 2022). For example, a broad scope supports the identification of the pandemic situation in the most critical regions to the business and breaks down the future impact into a finer level of detail.^5^

During the pandemic, managers are also concerned with communicating with stakeholders, particularly investors and analysts, about the strategic actions of the firm. Thus, managers signal to the market their response to the crisis by voluntarily revealing certain information and by observable strategic organizational decisions such as launching new products or services, implementing new strategies, or making new (dis)investments. The observability of those decisions spurs a positive market reaction that could potentially mitigate the effects of the crises. Accordingly, researchers have suggested that broad-scope MCS could support resilience thinking (i.e., the ability to resist and recover from unexpected events) (Oehmen et al. 2020; Spicer 2020)^6^.

Alternatively, some empirical evidence could undermine the potential benefits of broad-scope MCS in supporting managerial decision-making during a major pandemic. Studies on the relationship between the strategy and design of MCS found broad-scope MCS information to be particularly beneficial to organizations adopting a prospector rather than a defender strategy (Abernethy and Guthrie 1994). Those studies argue that broad-scope MCS are useful for prospectors as they aim for continuous product and market development and innovation but not for defenders, which aim to retain a comparatively stable market position. For defenders, a narrow-scope MCS, which is designed with an internal rather than external focus, is more appropriate because it enables them to maintain internal efficiencies. When faced with high levels of uncertainty, such as during crises, most organizations adopt a defender rather than a prospector strategy, as the objective is often to survive and be as efficient as possible with minimum resources (Collins et al. 1997). Additionally, managers using broad-scope MCS face the problem of needing to process a large volume of data (Garcia Osma et al. 2018). The information processing literature shows that limited attention is a common problem among decision-makers because they usually do not have the time or capacity to process all available information (Henri and Wouters 2020). The scope of learning in volatile and dynamic environments is inherently greater, as it requires managers to address concurrent priorities, which frequently raises problems of managerial bounded rationality and time constraints (Asel et al. 2011; García-Carbonell et al. 2021). In such circumstances, broad-scope MCS could overload decision-makers and distract them from the key issues that require their immediate attention. The lack of action or the externalization and filtering of contradictory or ill-formulated decisions could accelerate the negative market reaction that accompanies times of crisis.

Despite the arguments for a limited or possible negative effect of broad-scope MCS on the organizational response to the COVID-19 pandemic, we argue that, on balance, those MCS drive better and accurate decision-making that is likely to trickle to stakeholders and consequently plays a net mitigating role in the effects of the COVID-19 pandemic. Formally stated, our first hypothesis is as follows:

#### H1

Broad-scope MCS reduce the negative impact of a crisis with extreme uncertainty on shareholder expectations.

Boundary systems are an explicit set of organizational definitions and parameters that define the admissible space of activity for organizational participants (Simons 1995). They can be technical (e.g., budgetary limits) and/or social (e.g., code of conduct). Boundary systems allow managers the necessary flexibility to take action and make decisions while controlling behaviours by prescribing risks to avoid, activities considered acceptable and those that are not, and the domain where employees can search for new opportunities (Ferreira and Otley 2009; Heinicke et al. 2016; Garcia Osma et al. 2022).

In a setting of opportunity seeking such as a pandemic, the boundary system transmits risks to the entire organization and ensures that creative and innovative actions and solutions meet the demands of organizations even within predefined strategic limits. In this environment of uncertainty, where rapid actions to adapt to new market situations are needed, boundary controls allow subordinates to respond to local contingencies autonomously but within predefined boundaries (Simons 1995).

In a highly uncertain setting such as that of COVID-19, a clear definition of these acceptable and unacceptable experimentation activities and opportunity-seeking behaviours may play a role in our model, enhancing the effectiveness of the organizations’ responses and decisions (e.g., launching new products or services, implementing new strategies, or making new (dis)investments). Therefore, we investigate whether organizations with high levels of boundary systems are more likely to make within-bound decisions and actions and consequently obtain better market returns.

#### H2

The effect of broad-scope MCS reducing the negative impact of a crisis with extreme uncertainty on shareholder expectations is concentrated in firms with higher levels of boundary systems.

## Empirical design

### Sample and data

We test our hypotheses using data from B3, a stock exchange located in Sao Paulo, Brazil. COVID-19 is the deadliest virus in recent history, with a negative impact on global economic development. The latest WHO data^7^ show that Brazil ranks third among countries by the number of cases and deaths and that its economy has been heavily affected. Brazilian GDP decreased by 9% in the second quarter of 2020. The first market impacts were registered by the Stock Exchange (B3) in March 2020, when the Ibovespa index decreased by approximately 40%. This sudden crisis has prompted organizations to seek solutions, take action, and implement proper crisis management strategies. Beyond this institutional context, the multisectoral organizations in our sample are also an appropriate setting in which to test our hypotheses, as they are very large organizations and are thus more likely to present sophisticated and comprehensive MCS designs. Moreover, they have the necessary resources to potentially engage in popular but costly signaling methods such as social media.^8^

We use survey and archival data to test our hypotheses. Survey data were used to collect information about MCS scope and additional control variables at the organizational and managerial levels. The questionnaire was designed and administered following Dillman’s method and was sent in October 2019 to 655 senior managers (i.e., CEOs and CFOs). We obtained responses from 254 useable questionnaires (response rate of 38.8%), representing 168 individual organizations (39.7% of the B3 stock exchange).^9^ To check for potential nonresponse bias, we compared survey respondents with nonrespondents in terms of size. We also compared the answers provided by early and late respondents. This analysis yielded nonsignificant differences. We then matched the returned responses with weekly archival data from the Refinitiv Eikon database to measure market value before and after the declaration of the COVID pandemic in March 2020 by the WHO. Thus, a detailed panel data set was generated from the Eikon database for the period from July 2019 to July 2020 (52 weeks). Organization week is our unit of analysis for the dependent variable. This process generated 6,257 data points after excluding missing values.

### Measurement of key variables

#### Broad-scope MCS

We use a validated instrument to measure a broad-scope MCS (Chenhall and Morris 1986; Anzilago et al. 2022). This measure reflects the availability of four features of an organization’s MCS: (i) noneconomic information, such as customer preferences, employee attitudes, or competitive threats; (ii) external information, such as economic conditions or technological developments; (iii) nonfinancial production information, such as output rates, machine efficiency or employee absenteeism; and (iv) nonfinancial market information, such as market size or growth market share. While narrow-scope MCS focus on the past and on financial information, broad-scope MCS emphasize long-term, nonfinancial and financial data and both internal and external information. Factor analysis demonstrates that our measure is unidimensional (loadings in the 0.800–0.839 range) and explains 67.90% of the variance. Cronbach’s alpha is 0.842.

#### Boundary systems

We also use a validated instrument to measure boundary systems (Widener 2007; Heinicke et al. 2016; Anzilago et al. 2022). This instrument relies on four questions that ask respondents to specify the firm’s use of a code of business conduct and controls that communicate actions and decisions to be avoided (on a seven-point Likert scale): (i) appropriate behaviours; (ii) off-limit behaviours; (iii) risks to be avoided; and (iv) workforce awareness of the code of conduct. Factor analysis reveals that our measure is unidimensional (loadings in the 0.748–0.818 range) and explains 72.81% of the variance, while Cronbach’s alpha is 0.885.

#### Market value

Given that we analyse a multi-industry sample of large organizations, we employ a widely applicable measure of financial performance: market value to EBITDA (logarithm). The market value to EBITDA ratio (hereafter market value) is a valuation measure that reflects investors’ expectations and judgements about the quality of an organization’s future performance (Kabanoff and Brown 2008; Florio and Leoni 2017). The interpretation of this ratio is similar in nature to the interpretation of the price-to-earnings ratio (PER), but using EBITDA as the denominator, a less manageable figure via accruals, allows the evaluation of organizational performance without needing to factor in accounting and financial decisions or tax environments (Hawn et al. 2018).

### Empirical models

We estimate the following Eq. (1) to test H1:

1 $$\begin{gathered} {\text{Market}}\;{\text{value}} = \beta_{0} + \, \beta_{1} {\text{COVID-19}}\;{\text{pandemic}} + \beta_{2} {\text{broad-scope}}\;{\text{MCS}} \hfill \\ + \, \beta_{3} {\text{COVID-19}}\;{\text{pandemic}}*{\text{broad-scope}}\;{\text{MCS}} + \sum {\beta_{j} {\text{Controls}}} + \varepsilon \hfill \\ \end{gathered}$$

where market value is the dependent variable and the COVID-19 pandemic (as proxy for crisis with extreme uncertainty) and broad-scope MCS are our main independent variables. The COVID-19 pandemic is our treatment variable and equals 1 in weeks 33 to 52 and 0 otherwise. Week 33 (March 11, 2020) was the declaration of the COVID pandemic by the WHO. *β*_*3*_ is our coefficient of interest. This interaction, as expressed, tests our prediction that a broad-scope MCS mitigates the negative effect of the COVID-19 pandemic on market value. To test H2, we run Eq. (1) and split our sample using the mean value of boundary systems.

Based on prior literature, we include variables in Eq. (1) as controls for other factors that may be correlated with broad-scope MCS or associated with market value. In this regard, we include controls for additional MCS information characteristics (Chenhall and Morris 1986) to take into account the potential relevance of MCS design (Guenther and Heinicke 2019). We use three self-rated items (Likert scales) to measure each individual feature: timeliness (automatic receipt, frequency of reporting, and immediate reporting); aggregation (sectional reports, temporal reports, and decision models); and integration (subunit interaction, precise targets, and organizational effects). Overall, the results of the measurement analyses were satisfactory. We also control for the number of registered patents, family ownership (1 for a family and 0 otherwise), market culture (six Likert scale items: results-oriented, manager leadership, competitiveness, goal accomplishment, goal achievement, and market leadership) (Cameron and Quinn 2011; Spicer 2020), number of employees (log) and environmental complexity (two Likert scale items: diversity of product requirements and diversity of competitor strategies) (Bedford and Malmi 2015). Furthermore, we control for individual-level variables such as gender (1 for female and 0 otherwise), age (years) and tenure (years) (Bobe and Kober 2020). Finally, we include industry fixed effects with the one-digit code for NACE classification. We cluster standard errors at the week level.

Table 1 displays the descriptive statistics, which overall are consistent with those in previous studies, while Table 2 reports the measurement properties. Overall, these measurement analyses were satisfactory^10^. Table 3 shows the pairwise correlations. Similar to prior literature on market reactions to crises, the correlation between the COVID-19 pandemic and market value is negative. In addition, only correlations among MCS information characteristics and boundary systems are above 0.6. In untabulated results, we calculate the variance inflation factors (VIFs) to account for multicollinearity problems. Since the VIFs obtained are below the suggested cut-off value of 10, our model is not unduly influenced by multicollinearity.

**Table 1:** Descriptive statistics

	Mean	S.D	Min.	Q1	Median	Q3	Max.
Market value	2.343	0.851	− 2.340	1.845	2.205	2.682	6.095
Broad-scope MCS	5.572	0.996	2.250	5.250	6.000	6.500	7.000
Timeliness MCS	5.710	1.106	1.000	5.000	6.000	6.667	7.000
Aggregation MCS	5.845	0.985	2.000	5.333	6.000	6.667	7.000
Integration MCS	5.684	1.049	1.670	5.000	6.000	6.333	7.000
Boundary systems	5.851	0.988	2.250	5.500	6.000	6.500	7.000
Patents	40.34	175.260	0.000	0.000	0.000	8.000	1,570
Family	0.294	0.456	0.000	0.000	0.000	1.000	1.000
Market culture	5.772	0.935	1.000	5.500	6.000	6.333	7.000
Employees	5567.699	9,075.536	50.000	900.000	2000.000	7000.000	60,000.000
Environmental complexity	5.954	1.173	1.000	5.500	6.000	7.000	7.000
Gender	0.216	0.411	0.000	0.000	0.000	0.000	1.000
Age	40.405	8.277	26.000	35.000	39.000	46.000	64.000
Tenure	5.544	5.126	0.250	2.000	4.000	7.000	31.000

**Table 2:** Measurement properties

	Factor loadings (range)	Eigenvalues	Percentage variance explained	Cronbach’s alpha
Market value	–	–	–	–
Broad-scope MCS	0.800–0.839	2.716	67.90%	0.842
Timeliness MCS	0.851–0.872	2.231	74.38%	0.826
Aggregation MCS	0.859–0.874	2.244	74.81%	0.830
Integration MCS	0.780–0.821	1.897	63.22%	0.701
Boundary systems	0.748–0.818	2.912	72.81%	0.885
Patents	–	–	–	–
Family	–	–	–	–
Market culture	0.817–0.872	4.186	69.77%	0.911
Employees	–	–	–	–
Environmental complexity	0.938–0.938	1.759	87.97%	0.862
Gender	–	–	–	–
Age	–	–	–	–
Tenure	–	–	–	–

**Table 3:** Pearson correlation coefficients

		(1)	(2)	(3)	(4)	(5)	(6)	(7)
(1)	Market value	1.000						
(2)	COVID-19 pandemic	− 0.025	1.000					
(3)	Broad-scope MCS	− 0.088	0.006	1.000				
(4)	Timeliness MCS	− 0.024	0.001	0.756	1.000			
(5)	Aggregation MCS	− 0.091	0.003	0.754	0.779	1.000		
(6)	Integration MCS	0.022	0.003	0.754	0.790	0.747	1.000	
(7)	Boundary systems	0.083	0.001	0.640	0.572	0.567	0.617	1.000
(8)	Patents	− 0.046	− 0.001	0.175	0.132	0.131	0.129	0.140
(9)	Family	− 0.124	0.009	0.037	0.007	0.030	0.034	0.031
(10)	Market culture	− 0.045	− 0.003	0.590	0.709	0.524	0.623	0.582
(11)	Employees	0.070	− 0.005	0.120	− 0.009	− 0.031	− 0.041	0.049
(12)	Environmental complexity	− 0.002	0.001	0.480	0.490	0.445	0.482	0.456
(13)	Gender	0.059	− 0.011	− 0.007	0.055	− 0.036	− 0.007	0.022
(14)	Age	− 0.063	0.016	0.186	0.243	0.216	0.288	0.212
(15)	Tenure	− 0.234	0.013	0.111	0.220	0.206	0.264	0.232

		(8)	(9)	(10)	(11)	(12)	(13)	(14)
(8)	Patents	1.000						
(9)	Family	0.051	1.000					
(10)	Market culture	0.143	0.054	1.000				
(11)	Employees	0.108	− 0.121	− 0.035	1.000			
(12)	Environmental complexity	0.121	0.065	0.514	− 0.037	1.000		
(13)	Gender	0.058	− 0.030	0.077	− 0.161	0.052	1.000	
(14)	Age	0.042	0.112	0.177	− 0.040	0.042	− 0.189	1.000
(15)	Tenure	0.019	0.173	0.184	− 0.060	0.102	− 0.195	0.538

Coefficients greater than |0.019| are significant at the 10% level

## Results

### Main results

Table 4 displays the regression results to test H1. Consistent with prior assumptions, the effect of the COVID-19 pandemic on market value is negative and significant across models. The interaction term between the COVID-19 pandemic and broad-scope MCS is also positive and significant across models, providing support H1 and suggesting a mitigating effect of broad-scope MCS on the negative effect of the COVID-19 pandemic on market value. We have no ex-ante arguments about the direct effect of broad-scope MCS on market value. In isolation, our results show that the effect of firms using broad-scope MCS is negative. This finding is potentially in line with prior work suggesting that capital providers under certain conditions prefer narrower in scope and focused firms (Garcia Osma et al. 2018; Bilinski 2022). This potential information overload, which can distract managers, can be perceived negatively by investors. Taken together, our results suggest that in times of extreme uncertainty, broad-scope MCS are useful in signaling alternative paths, mitigating the potential market’s perception of less focused firms. Under such conditions, it seems that investors attach more weight to signals when the drivers of financial performance are included in the signal (i.e., consistent with the information provided by broad-scope MCS). This information allows investors to assess the persistence of firms’ performance (Ertimur et al. 2003).

**Table 4:** The effect of Broad-scope MCS reducing the negative impact of COVID-19 pandemic on Market value (H1)

	Market value Coef. (S.E.)			
	(1)	(2)	(3)	(4)
Intercept	1.377 (0.059)***	1.380 (0.059)***	1.502 (0.068)***	6.962 (0.163)***
COVID-19 pandemic	− 0.035 (0.021)*	− 0.033 (0.021)*	− 0.179 (0.001)***	− 0.120 (0.001)***
Broad-scope MCS	− 0.310 (0.011)***	− 0.361 (0.013)***	− 0.364 (0.013)***	− 0.930 (0.024)***
COVID-19 pandemic*Broad-scope MCS		0.140 (0.023)***	0.143 (0.023)***	0.136 (0.023)***
COVID-19 pandemic*Timeliness MCS		− 0.088 (0.021)***	− 0.090 (0.021)***	− 0.108 (0.018)***
COVID-19 pandemic*Aggregation MCS		− 0.043 (0.011)***	− 0.045 (0.011)***	− 0.070 (0.008)***
COVID-19 pandemic*Integration MCS		− 0.026 (0.008)***	− 0.025 (0.008)***	− 0.004 (0.004)
Timeliness MCS	0.020 (0.016)	0.052 (0.020)**	0.053 (0.020)**	− 0.230 (0.028)***
Aggregation MCS	− 0.023 (0.005)***	− 0.008 (0.006)	− 0.006 (0.005)	0.657 (0.020)***
Integration MCS	0.273 (0.005)***	0.284 (0.005)***	0.282 (0.005)***	0.630 (0.020)***
Patents	0.000 (0.000)	0.000 (0.000)	0.000 (0.000)	0.000 (0.000)***
Family	− 0.153 (0.013)***	− 0.154 (0.013)***	− 0.155 (0.013)***	0.766 ((0.041)***
Market culture	− 0.097 (0.003)***	− 0.097 (0.004)***	− 0.096 (0.004)***	0.379 (0.014)***
Employees	0.051 (0.004)***	0.051 (0.005)***	0.051 (0.005)***	− 0.295 (0.006)***
Environmental complexity	0.087 (0.003)***	0.087 (0.003)***	0.087 (0.003)***	− 0.198 (0.008)***
Gender	− 0.046 (0.009)***	− 0.047 (0.009)***	− 0.047 (0.009)*	− 0.796 (0.055)***
Age	0.012 (0.000)***	0.012 (0.000)***	0.012 (0.000)***	0.006 (0.002)**
Tenure	− 0.052 (0.001)***	− 0.052 (0.001)***	− 0.051 (0.001)***	− 0.309 (0.004)***
Industry fixed effects	Yes	Yes	Yes	Yes
Week fixed effects	No	No	Yes	Yes
Firm fixed effects	No	No	No	Yes
Observations	6257	6257	6257	6257
R^2^	0.267	0.269	0.281	0.921

∗ , ∗  ∗ , ∗  ∗  ∗ indicate significance at the 10%, 5%, and 1% levels (two-tailed (one-tailed) tests are presented for non-directional (hypothesized directional) expectations), respectively. Standard errors are adjusted for clustering at the week level

Although in our main results we include a set of variables to control for firm-level features, one potential concern is that our results may be driven by correlated omitted variables at the firm level. To mitigate this concern, we run our main model controlling for firm fixed effects.^11^ The results reported in Table 4 (model 4) show that our findings remain robust (COVID-19 pandemic*Broad-scope MCS, *β* = 0.136; *p* < 0.01).

In untabulated sensitivity results, we also ran our model using an alternative definition of the COVID-19 pandemic variable (using week 45, when Brazil counted 10,000 infected cases). The results were qualitatively similar to those reported using week 33 as the treatment. We also rerun our analysis after using market value as the dependent variable instead of the ratio and continue to find results (untabulated). Overall, our results are robust to these alternative specifications.

Our main premise rests on the markets detecting a firm's signals. A question that emerges is whether other major providers of capital, such as lenders, are also detecting these signals. To analyse this effect, we run our analyses using the firm cost of debt as the dependent variable.^12^ Consistent with other reported evidence (e.g., Deloitte 2021), we find that firms equipped with broad-scope MCS are more likely to decrease the cost of debt in times of economic crisis (see Table 5: *β* = − 0.178, *p* < 0.01).

**Table 5:** The effect of Broad-scope MCS reducing the negative impact of COVID-19 pandemic on Cost of debt (H1)

	Cost of debt	
	(1)	(2)
COVID-19 pandemic	0.011 (0.812)	− 0.025 (− 1.522)*
Broad-scope MCS	− 0.252 (− 5.701)***	− 0.142 (− 3.061)***
COVID-19 pandemic*Broad-scope MCS		− 0.178 (− 3.577)***
COVID-19 pandemic*Timeliness MCS		− 0.316 (− 6.470)***
COVID-19 pandemic*Aggregation MCS		0.430 (6.083)***
COVID-19 pandemic*Integration MCS		− 0.021 (− 0.538)
Timeliness MCS	0.062 (1.034)	0.267 (3.508)***
Aggregation MCS	− 0.040 (− 0.376)	− 0.321 (− 2.426)**
Integration MCS	0.180 (3.714)***	0.195 (3.089)***
Patents	0.106 (4.665)***	0.108 (4.835)***
Family	− 0.101 (− 8.789)***	− 0.101 (− 8.742)***
Market culture	− 0.157 (− 4.597)***	− 0.149 (− 4.901)***
Employees	− 0.126 (− 6.295)***	− 0.125 (− 6.389)***
Environmental complexity	− 0.019 (− 1.137)	− 0.012 (− 0.738)
Gender	− 0.034 (− 1.186)	− 0.033 (− 1.208)
Age	0.116 (6.564)***	0.112 (6.584)***
Tenure	− 0.055 (− 3.269)***	− 0.056 (− 3.353)***
Industry fixed effects	Yes	Yes
Week fixed effects	Yes	Yes
Observations	3159	3159
R^2^	0.071	0.134

∗ , ∗  ∗ , ∗  ∗  ∗ indicate significance at the 10%, 5%, and 1% levels (two-tailed (one-tailed) tests are presented for non-directional (hypothesized directional) expectations), respectively. T statistics based on robust standard errors are reported in parentheses. Standardized coefficients are displayed

We split our sample between high and low levels of boundary systems to test H2. The results in Table 6 show that the positive effect of broad-scope MCS under COVID-19 is concentrated in the subsample with high boundary systems (COVID-19 pandemic*Broad-scope MCS, *β* = 0.259; *p* < 0.01), as expected. Moreover, for organizations that are not classified as having high boundaries, we find evidence of a negative association between the COVID-19 pandemic*Broad-scope MCS and market value (*β* = − 0.033; *p* < 0.01). We interpret these findings as indicating that boundary systems focus and filter out strategic actions, ensuring that managers engage in functional initiatives and avoid superfluous actions. Our results provide evidence that in a pandemic and an uncertain context, limit control ensures that the freedom granted to managers to take action and decision-making is restricted within certain preestablished limits and that managers do not become confused about actions without short-term viability. These focused actions increase investors’ expectations and judgements about the quality of an organization’s future performance.

**Table 6:** The effect of Broad-scope MCS reducing the negative impact of COVID-19 pandemic on Market value by Boundary systems (H2)

	Market value Coef. (S.E.)			
	Low Boundary systems subsample		High Boundary systems subsample	
Intercept	0.033 (0.054)	0.016 (0.053)	1.736 (0.132)***	1.733 (0.130)***
COVID-19 pandemic	− 0.069 (0.000)***	− 0.025 (0.004)***	0.012 (0.001)***	0.025 (0.009)***
Broad-scope MCS	− 0.024 (0.007)***	− 0.226 (0.008)***	− 0.329 (0.027)***	− 0.428 (0.029)***
COVID-19 pandemic*Broad-scope MCS		− 0.033 (0.009)***		0.259 (0.046)***
COVID-19 pandemic*Timeliness MCS		− 0.062 (0.011)***		− 0.068 (0.044)
COVID-19 pandemic*Aggregation MCS		0.025 (0.008)***		− 0.096 (0.030)***
COVID-19 pandemic*Integration MCS		0.129 (0.015)***		− 0.138 (0.022)***
Timeliness MCS	0.210 (0.009)***	0.231 (0.011)***	0.184 (0.036)***	0.208 (0.041)***
Aggregation MCS	− 0.023 (0.012)*	− 0.035 (0.014)**	− 0.086 (0.016)***	− 0.045 (0.015)***
Integration MCS	0.221 (0.013)***	0.173 (0.010)***	0.157 (0.015)***	0.207 (0.008)***
Patents	0.008 (0.000)***	0.008 (0.000)***	− 0.000 (0.000)***	− 0.000 (0.000)***
Family	0.043 (0.017)**	0.041 (0.017)**	− 0.107 (0.026)***	− 0.109 (0.026)***
Market culture	− 0.193 (0.007)***	− 0.195 (0.007)***	− 0.088 (0.012)***	− 0.083 (0.011)***
Employees	0.117 (0.003)***	0.116 (0.003)***	0.061 (0.008)***	0.060 (0.008)***
Environmental complexity	0.151 (0.014)***	0.152 (0.014)***	0.093 (0.007)***	0.094 (0.007)***
Gender	0.411 (0.039)***	0.414 (0.039)***	− 0.205 (0.022)***	− 0.208 (0.022)***
Age	0.039 (0.002)***	0.039 (0.001)***	0.000 (0.001)	0.000 (0.001)
Tenure	− 0.103 (0.004)***	− 0.104 (0.004)***	− 0.052 (0.002)***	− 0.052 (0.002)***
Industry fixed effects	Yes	Yes	Yes	Yes
Week fixed effects	Yes	Yes	Yes	Yes
Observations	2297	2297	3,960	3960
R^2^	0.597	0.600	0.301	0.307

∗ , ∗  ∗ , ∗  ∗  ∗ indicate significance at the 10%, 5%, and 1% levels (two-tailed (one-tailed) tests are presented for non-directional (hypothesized directional) expectations), respectively. Standard errors are adjusted for clustering at the week level

These results can also be explained by the fact that in the COVID-19 pandemic, in addition to economic challenges, organizations needed to attend the health crisis. In this scenario, companies’ social responsibility played a fundamental role in guaranteeing the safety and adequate organization of employees and, in turn, the other stakeholders. For example, organizations are required to effectively communicate policies about the protection of health and safety. Any changes to those policies should be communicated as early as possible. This situation boosted the visibility of the limits about strategic and operative responses to the pandemic. Therefore, organizational policies, especially those involving employees and customers, were immediately noticed by investors. As a useful example, Brazilian slaughterhouses had to cope with delays in the supply of raw materials, logistical issues or changes in customer demands. In response, they implemented strategic and operational measures that avoided interruptions in the supply of meat to consumers (Infomoney 2021). However, most of these companies were penalized with drops in market value for signaling with references only to financial information (i.e., narrow scope) as they traditionally did. If they would have included the drivers of that financial performance (i.e., broad scope information), investors could have assessed the persistence of the firms’ financial performance (Ertimur et al. 2003). This outcome is in line with our findings, suggesting that in a pandemic environment, broad-scope MCS may not be effective without the presence of boundary systems.

### Additional results: alternative empirical identification

The evidence presented in previous section suggests that broad-scope MCS mitigate the negative effect of the COVID-19 pandemic on market value. Our primary identification strategy exploits the declaration of the COVID pandemic in March 2020 by the WHO, providing a time-variant testing ground for our research question. In this section, we run additional sensitivity checks by empirically analysing whether our findings supporting H1 and H2 persist using an alternative identification: the number of new COVID-19 deaths in Brazil (at the week level). The results in Tables 7 and 8 are generally consistent with those reported in Tables 4 and 6, providing additional robustness for the main findings. However, while in Table 7 the effect of new COVID-19 deaths on market value is negative and significant, in splitting the sample this effect is not significant. This difference in the effect could be because this proxy does not fully capture the full number of events that impacted the markets during those weeks. Overall, these findings provide confidence that it is the COVID-19 context that drives our results, not potential spurious consequences from time-fixed effects.

**Table 7:** The effect of Broad-scope MCS reducing the negative impact of COVID-19 pandemic on Market value. Alternative empirical identification

	Market value Coef. (S.E.)	
	(1)	(2)
New COVID-19 deaths	− 0.063 (0.000)*	− 0.062 (0.000)*
Broad-scope MCS	− 0.328 (0.019)***	− 0.352 (0.022)***
New COVID-19 deaths*Broad-scope MCS		0.049 (0.000)**
New COVID-19 deaths*Timeliness MCS		− 0.028 (0.000)
New COVID-19 deaths*Aggregation MCS		− 0.009 (0.000)
New COVID-19 deaths*Integration MCS		− 0.019 (0.000)
Timeliness MCS	0.022 (0.026)	0.035 (0.028)
Aggregation MCS	− 0.025 (0.018)	− 0.020 (0.020)
Integration MCS	0.315 (0.017)***	0.324 (0.020)***
Patents	0.004 (0.000)	0.004 (0.000)
Family	− 0.081 (0.020)***	− 0.081 (0.020)***
Market culture	− 0.114 (0.013)***	− 0.113 (0.013)***
Employees	0.089 (0.008)***	0.089 (0.008)***
Environmental complexity	0.109 (0.011)***	0.109 (0.010)***
Gender	− 0.022 (0.027)*	− 0.022 (0.009)*
Age	0.110 (0.001)***	0.111 (0.001)***
Tenure	− 0.313 (0.002)***	− 0.313 (0.002)***
Industry fixed effects	Yes	Yes
Week fixed effects	Yes	Yes
Observations	6257	6257
R^2^	0.279	0.280

∗ , ∗  ∗ , ∗  ∗  ∗ indicate significance at the 10%, 5%, and 1% levels (two-tailed (one-tailed) tests are presented for non-directional (hypothesized directional) expectations), respectively. Robust standard errors in parentheses. Standardized coefficients are displayed

**Table 8:** The effect of Broad-scope MCS reducing the negative impact of COVID-19 pandemic on Market value by Boundary systems. Alternative empirical identification

	Market value Coef. (S.E.)			
	Low Boundary systems subsample		High Boundary systems subsample	
New COVID-19 deaths	− 0.001 (0.000)	0.027 (0.000)	0.008 (0.000)	0.008 (0.000)
Broad-scope MCS	− 0.336 (0.016)***	− 0.322 (0.017)***	− 0.227 (0.040)***	− 0.250 (0.045)***
New COVID-19 deaths*Broad-scope MCS		− 0.024 (0.000)		0.058 (0.000)**
New COVID-19 deaths*Timeliness MCS		− 0.046 (0.000)		0.018 (0.000)
New COVID-19 deaths*Aggregation MCS		0.007 (0.000)		− 0.003 (0.000)
New COVID-19 deaths*Integration MCS		0.118 (0.000)***		− 0.084 (0.000)***
Timeliness MCS	0.301 (0.028)***	0.321 (0.030)***	0.128 (0.041)***	0.118 (0.045)***
Aggregation MCS	− 0.034 (0.023)	− 0.040 (0.026)	− 0.064 (0.032)***	− 0.062 (0.033)**
Integration MCS	0.323 (0.015)***	0.269 (0.017)***	0.124 (0.034)***	0.161 (0.038)***
Patents	0.405 (0.000)***	0.399 (0.000)***	− 0.053 (0.000)***	− 0.053 (0.000)***
Family	0.024 (0.026)*	0.023 (0.026)	− 0.055 (0.029)***	− 0.055 (0.028)***
Market culture	− 0.332 (0.014)***	− 0.335 (0.014)***	− 0.049 (0.029)***	− 0.048 (0.028)***
Employees	0.239 (0.010)***	0.238 (0.016)***	0.097 (0.012)***	0.096 (0.011)***
Environmental complexity	0.237 (0.016)***	0.238 (0.014)***	0.089 (0.015)***	0.090 (0.015)***
Gender	0.168 (0.052)***	0.169 (0.052)***	− 0.098 (0.034)***	− 0.099 (0.033)***
Age	0.459 (0.002)***	0.461 (0.002)***	0.002 (0.002)	0.002 (0.002)
Tenure	− 0.324 (0.007)***	− 0.325 (0.007)***	− 0.349 (0.003)***	− 0.348 (0.003)***
Industry fixed effects	Yes	Yes	Yes	Yes
Week fixed effects	Yes	Yes	Yes	Yes
Observations	2297	2297	3960	3960
R^2^	0.597	0.600	0.301	0.304

∗ , ∗  ∗ , ∗  ∗  ∗ indicate significance at the 10%, 5%, and 1% levels (two-tailed (one-tailed) tests are presented for non-directional (hypothesized directional) expectations), respectively. Robust standard errors in parentheses. Standardized coefficients are displayed

## Discussion and conclusions

This study draws on both signaling theory and the management accounting and crisis management literature to examine the extent to which a broad-scope MCS mitigates the negative impact of COVID-19 on an organization’s market value. Based on an original survey and weekly archival data retrieved from Thomson Reuters’ Eikon database, our findings suggest that broad-scope MCS indeed mitigate the negative impact of COVID-19 on organizations’ market value. Framing our study with signaling theory, we illustrate the benefits of this framework to management accounting research. Thus, the power of signaling theory lies in making predictions using single behavioural postulations (e.g., rational parties). Starting with the assumption that managers use this accounting information to increase their breadth of vision in managerial decision-making, signaling theory offers a basis to understand how investors and, more broadly, markets respond to organizational movements under conditions of incomplete information in an unpredictable and unknown setting (e.g., COVID-19 pandemic).

In addition, we provide further insights into the role of boundary systems in our model. These results indicate that the positive effect of broad-scope MCS on market value is concentrated mainly in organizations with higher levels of boundary systems, suggesting that investors value the breadth of vision brought by broad-scope MCS when managers respond to the crisis within certain preestablished strategic boundaries. However, broad-scope MCS could divert and create ambiguity for organizations with low boundary systems.

This study sheds more light on the effectiveness of MCS during a major crisis. Compared to other crises (e.g., natural disasters or financial crises), COVID-19 is an exceptional situation where the level of uncertainty is amplified due to its rapid global spread across countries and markets. This research offers meaningful implications for managers regarding information gathering and the market value consequences of their decisions in a dismal environment with vast uncertainty. Future research could examine the role of MCS in coping with other challenges derived from dramatic and unexpected events, such as the tension generated when managing hostility and uncertainty (Otley 2016). That is, we need to learn how organizations design and use MCS to overcome the uncertainty imposed but also to be more agile, flexible and innovative to cope with market hostility (Gomez-Conde et al. 2021). Following this argumentation, we see some avenues for further research. First, using this categorization of MCS design, subsequent work can further disentangle how other dimensions of MCS (e.g., integration, timeliness, and aggregation) affect decision-making in this unpredictable setting. Second, although this paper focuses on market value and investor perception, broad-scope MCS influence many other areas of decision-making and control in an organization, such as strategy reformulation, capital investment decisions or innovation processes. New insights could deepen the understanding of MCS on the quality and accuracy of these decisions and their potential outcomes in a pandemic. We look forward to new studies that offer answers to these important questions.

Our study contributes to prior work in the following ways. First, by analysing the effect of broad-scope MCS and a broad vision on firm market value, an important but overlooked research field, we bridge this gap between management accounting and financial markets (Davila et al. 2015; Hemmer and Labro 2019). Second, by using this exceptional setting, the worldwide COVID-19 pandemic, we advance existing research on the role of MCS in helping managers undertake more accurate decision-making in firms facing uncertainty, crises or environmental pressures (Conrad and Guven-Uslu 2012; Janke et al. 2014; Becker et al. 2016).

The results of this study are subject to limitations. First, this research relied on the recollections of survey respondents. We acknowledge the limitations of such a research approach and suggest that future research attempt to obtain archival data about the use of broad-scope MCS. Second, the conceptual background and data of MCS refer to information availability rather than the actual use and implementation thereof. We also recognize the limitation of missing important dimensions of MCS, such as attention patterns. Finally, although market value is used as a valid indicator of effective internal decision-making in prior work, we acknowledge the limitations of this measure in fully capturing the perceptions of internal management quality.

## Survey items

### Management control system design

Indicate your perception of the availability of the following features in your company's management control system: (Scale: 1-not at all to 7-to a great extent)

**Table:**

*Scope*	
1. Noneconomic information, such as customer preferences, employee attitudes, labour relations, attitudes of government and consumer bodies, competitive threats, etc.	
2. Information on broad factors external to your organization, such as economic conditions, population growth, technological developments, etc.	
3. Nonfinancial information that relates to production information such as output rates, scrap levels, machine efficiency, employee absenteeism, etc.	
4. Nonfinancial information that relates to market information such as market size, growth share, etc.	
*Aggregation*	
1. Information provided on the different sections or functional areas of your organization, such as marketing and production, or sales, cost, among others.	
2. Information on the effect of events on particular time periods (e.g., monthly/semiannual/annual summaries, trends, comparisons, etc.).	
3. Information in formats suitable for input into decision models such as discounted cash flow analysis, incremental or marginal analysis, credit policy analysis, etc.	
*Timeliness*	
1. Information supplied to you automatically upon its receipt into information systems or as soon as processing is completed.	
2. Reports are provided frequently on a systematic, regular basis:, e.g., daily reports, weekly reports (for less frequent reporting, mark lower end of scale).	
3. There is no delay between an event occurring and relevant information being reported to you	
*Integration*	
1. Information on the impact that your decision will have throughout your department, and the influence of other individuals' decisions on your area of responsibility.	
2. Precise targets for the activities of all sections within your department.	
3. Information that relates to the impact of your decisions on the performance of your department.	

### Environmental complexity

Indicate your perception (Scale: 1-low to 7-to high)

**Table:**

1. How diverse are the product/service requirements of your customers to each other?	
2. How diverse are the strategies and tactics of your key competitors to each other?	

### Market culture

**Table:**

*Indicate your perception* (*Scale: 1-not at all to 7-to a great extent*)	
1. The company is results-oriented. A major concern is with getting the job done. People are very competitive and achievement-orientated	
2. The leadership in the company is generally considered to exemplify a no-nonsense, aggressive, results-orientated focus	
3. The management style in the company is characterized by hard-driving competitiveness, high demand and achievement	
4. The ‘glue’ that holds the company together is the emphasis on achievement and goal accomplishment	
5. The company emphasizes competitive actions and achievement. Hitting stretch targets and winning in the marketplace are dominant	
6. The company defines success on the basis of winning in the marketplace and outpacing the competition. Competitive market leadership is the key	

### Boundary systems

Indicate your perception (Scale: 1-strongly disagree 7-strongly agree)

**Table:**

1. Our organization relies on a code of business conduct to define appropriate behaviour for our workforce	
2. Our code of business conduct informs our workforce about behaviours that are off-limits	
3. Our organization communicates to our workforce risks that should be avoided	
4. Our workforce is aware of the organization’s code of business conduct	

## Footnotes

1 Nichols and Wahlen (2022) highlight that nonfinancial firm-specific announcements, together with other sources such as financial press reports, might forestall financial information as sources of information for investors.

2 As an example, on 03/30/2020, a well-known electronic equipment firm in the Brazilian Stock Exchange reported the signature of a technology transfer agreement with other manufacturer of medical-hospital equipment to produce artificial ventilators (https://ri.weg.net/en/publications-and-documents/news). Petrobras (ranked #120 in the most recent Fortune Global 500 list) made numerous announcements to shareholders and the market about the main measures adopted to confront and mitigate the effects of COVID-19 on its operations (https://petrobras.com.br/fatos-e-dados/todos-juntos-contra-o-coronavirus.htm).

3 The European Securities and Market Authority (ESMA) recommends that firms include detailed information in their interim management reports closely associated with the information supplied by a broad-scope MCS. Among other measures, the ESMA recommends disclosing the following: “(a) The impact that the COVID-19 pandemic has had on their strategic orientation and targets, operations, […]; (b) Measures taken to address and mitigate the impacts of the COVID-19 pandemic on their operations and performance and their progress/state of completion […]; (c) The expected future impact on organizational performance and contingency measures planned to mitigate the expected future impact and risk and uncertainties identified […]”.

4 A potential concern is whether the pandemic enforces less broad-scope MCS. Although the adjustment of MCS due to external forces is plausible, it requires several periods to materialize (Garcia-Osma et al. 2022). We thank an anonymous reviewer for making this point.

5 Some potential KPIs in a broad-scope MCS are reduction in traffic congestion or the level of restaurant bookings and customer details (i.e., food preferences or allergies).

6 As an illustrative example, the collaboration of Inditex with the MIT has broadened the traditional internal focus of MCS based on historical data (i.e., the spending habits of customers) with external information captured via social media (Inditex 2019). This perspective “helped Inditex weather the fallout from the global lockdown better than any other retailers. […] already in March, Inditex had ramped up web discounts, a rare move for a firm that emphasizes how online and offline operations are always in sync” (Orihuela and Hipwell 2020).

7 By July 2022.

8 Bilinski (2022), for example, suggests that the cost of obtaining the information, shaping the message and eventually preparing audio-visual content for social media communication can be nontrivial. He estimates that promoting a trend on Twitter can cost $200,000 per day for a firm.

9 Eighty-six questionnaires were received from a second informant. We considered the first response received for each individual organization. As an additional untabulated sensitivity check, we ran our main model using all responses, yielding qualitatively similar results.

10 As in extant prior work (Mahlendorf et al. 2014; Garcia Osma et al. 2018; Anzilago et al. 2022), we use the following criteria: factor loadings > 0.5; eigenvalues > 1; percentages of variance explained > 50%, Cronbach’s alphas > 0.7.

11 We are grateful to one of the reviewers for this suggestion.

12 To run this test, we use available data of cost of debt in our sample: 3,159 firm-week observations. Cost of debt is the ratio of interest expenses divided by short-term debt.
